# Diffusion-weighted MRI characteristics of the cerebral metastasis to brain boundary predicts patient outcomes

**DOI:** 10.1186/1471-2342-14-26

**Published:** 2014-08-03

**Authors:** Rasheed Zakaria, Kumar Das, Mark Radon, Maneesh Bhojak, Philip R Rudland, Vanessa Sluming, Michael D Jenkinson

**Affiliations:** 1 Department of Neurosurgery, The Walton Centre NHS Foundation Trust, Liverpool, UK; 2 Institute of Integrative Biology, University of Liverpool, Liverpool, UK; 3 Department of Neuroradiology, The Walton Centre NHS Foundation Trust, Liverpool, UK; 4 Magnetic resonance and image analysis research centre (MARIARC), University of Liverpool, Liverpool, UK; 5 Institute of Translational Medicine, University of Liverpool, Liverpool, UK; 6 Neurosciences Research Centre, The Walton Centre NHS Foundation Trust, Liverpool L9 7LJ, UK

**Keywords:** Cerebral metastasis, Brain neoplasms, Secondary, Neoplasm invasiveness, Diffusion-weighted imaging, Apparent diffusion coefficient, MRI

## Abstract

**Background:**

Diffusion-weighted MRI (DWI) has been used in neurosurgical practice mainly to distinguish cerebral metastases from abscess and glioma. There is evidence from other solid organ cancers and metastases that DWI may be used as a biomarker of prognosis and treatment response. We therefore investigated DWI characteristics of cerebral metastases and their peritumoral region recorded pre-operatively and related these to patient outcomes.

**Methods:**

Retrospective analysis of 76 cases operated upon at a single institution with DWI performed pre-operatively at 1.5T. Maps of apparent diffusion coefficient (ADC) were generated using standard protocols. Readings were taken from the tumor, peritumoral region and across the brain-tumor interface. Patient outcomes were overall survival and time to local recurrence.

**Results:**

A minimum ADC greater than 919.4 × 10^-6^ mm^2^/s within a metastasis predicted longer overall survival regardless of adjuvant therapies. This was not simply due to differences between the types of primary cancer because the effect was observed even in a subgroup of 36 patients with the same primary, non-small cell lung cancer. The change in diffusion across the tumor border and into peritumoral brain was measured by the “ADC transition coefficient” or ATC and this was more strongly predictive than ADC readings alone. Metastases with a sharp change in diffusion across their border (ATC >0.279) showed shorter overall survival compared to those with a more diffuse edge. The ATC was the only imaging measurement which independently predicted overall survival in multivariate analysis (hazard ratio 0.54, 95% CI 0.3 – 0.97, p = 0.04).

**Conclusions:**

DWI demonstrates changes in the tumor, across the tumor edge and in the peritumoral region which may not be visible on conventional MRI and this may be useful in predicting patient outcomes for operated cerebral metastases.

## Background

Cerebral metastases are an increasingly common problem with an incidence ten times that of primary brain tumors and the diagnosis is often made by general radiologists [1,2]. Traditionally MRI has been used for assessing the site and number of metastases, planning for surgery or radiosurgery and assessing the response to therapy [3,4]. Newer MRI techniques have been applied to help distinguish solitary cerebral metastases from mimics such as high grade glioma or abscess. One common example is by measuring the ADC of the lesion but these readings have more recently been suggested to have some *prognostic* value as well as diagnostic [5,6]. When comparing cerebral metastases to glioma considerable attention has focused on the peritumoral region around the lesion [7-14]. A number of metrics from DWI including ADC values have been directly measured in this region and the “normal” range of values for a metastasis determined [15]. The interaction of cerebral metastases and the surrounding brain tissue would be expected to be crucial to the development of therapies aimed at preventing further spread and invasion. Work on different methods of invasion - either co-opting existing blood vessels or inducing new blood vessel formation - has already led to anti-angiogenesis drugs for certain cerebral metastases [16,17]. Furthermore, the degree of invasion of metastases may affect the margin used in existing treatments such as surgery and radiosurgery. Despite this potential clinical importance little has been done to investigate the invasiveness of cerebral metastases using MRI and nobody has compared the tumor and peritumoral ADC readings *within* a group of metastases. We therefore examined the MRI characteristics of the tumor, tumor boundary and peritumoral region for a series of patients with cerebral metastases who had undergone pre-operative MRI with DWI followed by surgery. We investigate whether patient outcomes such as survival and recurrence may be predicted by different DWI metrics.

## Methods

### Patients

Patients with a diagnosis of cerebral metastasis were identified from a histopathology archive spanning the period 2007 – 2012 at a single institution. Records were searched and cases with a diffusion-weighted MRI scan of the brain prior to first neurosurgical intervention were selected. Recursive Partitioning Analysis (RPA) [18] class and Graded Prognostic Assessment (GPA) [19] score were determined; these are validated predictive measures in cerebral metastasis patients and are based on information such as control of the primary cancer, extra-cranial metastases, number of metastases and general health or “performance status” of the patient. Post-operative clinical course and oncology care including administration of whole brain radiotherapy (WBRT) or the use of adjuvant systemic chemotherapy were recorded from tumor board and patient notes as these were potential confounding factors. This study was conducted in accordance with the principles of the declaration of Helsinki and ethical approval was granted as an internal project within the institution’s research tissue bank (National Research Ethics Service # 11/WNo03/2).

### Histological analysis

All cases had been reviewed by a consultant neuropathologist in the course of routine clinical care. A histological diagnosis of metastasis was made and primary tumor type suggested by review of H & E sections, tumor specific immuno-histo-cytochemistry and clinical correlation. Where original material was available an additional automated assessment of cellularity was then made “blind” to the clinical and imaging data. Five separate 200× magnification fields were photographed avoiding gross haemorrhage or necrotic material on the H & E slide. Using Image J software (U. S. National Institutes of Health, Bethesda, Maryland, USA 2007-2012) with no additional plugins the percentage nuclear area in each field was determined using the measure function and a mean taken. A second independent observer, a cellular pathology researcher of 30 years’ experience then repeated this analysis manually using a grid and microscopy.

### MRI acquisition and analysis

All patients had undergone preoperative MRI brain scans on different whole body systems at 1.5T with a single channel head coil at local institutions before transfer to the Regional Neuroscience Centre (of the 76 cases: 11 on GE Signa HD, 6 on a GE Discovery, 43 on Philips Achieva and 16 on a Siemens Avanto). All patients had received at least 24-48 hours of dexamethasone treatment (of at least 4mg twice daily orally) prior to MR imaging. The imaging included DWI using one acquisition over 90 seconds through single-shot echo planar imaging with two b values of 0 and 1000 second/mm^2^ in all cases. MR parameters were similar between scanners and in the following range: slice thickness 6mm for all, TR 2515 - 3513 msec, TE 71-94 msec. Trace-weighted imaging and ADC trace maps were calculated and subsequent readings taken using the post processing software package GE FuncTool version 4.5.5 (General Electric Co., Maryland, USA). All patients had also undergone a fast spoiled gradient echo (FSPGR) or equivalent sequence with gadolinium contrast, again variable between institutions but most commonly TR 25 msec, TE 6.1 msec, flip angle 20 degrees.

**Figure 1:**
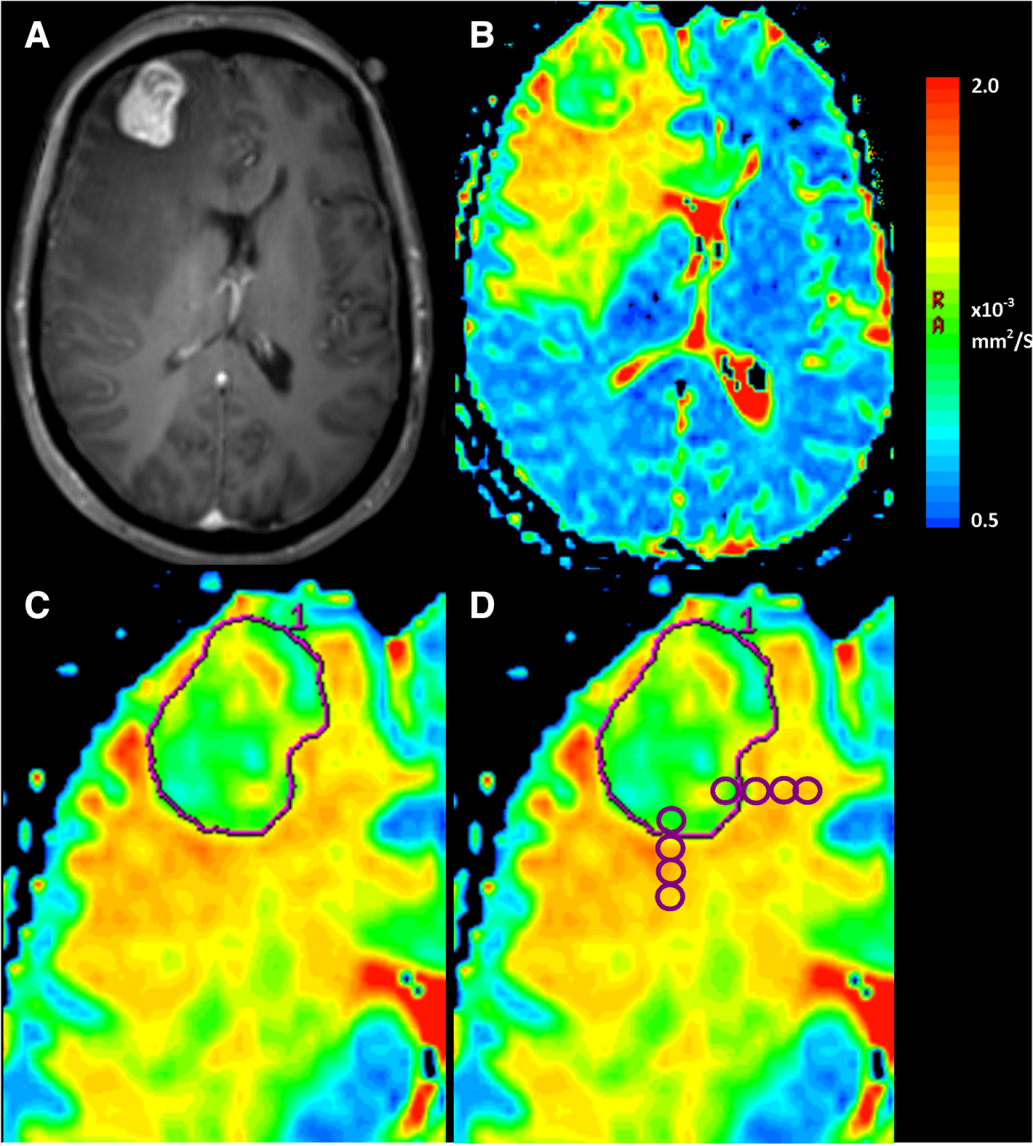
**Measurement of ADC metrics from brain metastases on diffusion-weighted MRI scans.** A patient with a history of lung adenocarcinoma presents with headache and focal neurological deficit. **A**. Postcontrast T1-weighted sequence demonstrates a right frontal lesion, which was confirmed as a metastasis after excision. **B**. An ADC map is generated from B = 0.1000 images using post processing software. On this color map, blue areas represent low ADC and red higher ADC. **C**. A freehand region of interest is traced around the tumor border using the postcontrast T1-weighted sequence as reference on the axial slice with the largest tumor area and those immediately above and below. The ADCmin is the lowest bin of a histogram of ADC values for all pixels contained within this ROI, averaged over these three slices. Three ROI are placed within the tumor on the axial slice with the largest area, avoiding necrosis, haemorrhage, cyst and the mean taken to give ADCmean. **D**. To assess change of ADC across the tumor border, four ROI are placed starting just inside the tumor border and extending out into the peritumoral region. The slope of the four ADC values is taken as the “ADC transition coefficient” or ATC. This is repeated in 3 orthogonal directions, avoiding structures such as ventricle or falx and then repeated on two slices above and below, with the ATC being the mean of the 9 readings.

Conventional MRI measures were taken on this postcontrast T1-weighted sequence including the number of lesions, maximum diameter on axial slices and volume (using three orthogonal measurements of diameter). The largest, operated metastasis only was assessed for those with multiple lesions (note that in general the cases with more than one metastasis included a dominant lesion with only small additional lesions). A number of different assessments of diffusion were made for each metastasis from the DWI scan and resulting ADC map. Figure 1 demonstrates the position of regions of interest (ROI) for recording these different ADC metrics. We have previously reported excellent intra and inter-observer reliability taking the readings below on this software for 12 of the 76 patients described, with one observer a clinical research fellow in neurosurgery and the second a clinician of 10 years specialised training in neuroradiology [20].

• **ADCmin**: The axial slice with the largest area of tumor was identified and a freehand ROI created using the tumor border on postcontrast T1-weighted sequence. This was repeated on the slices immediately above and below using the same method. The software then gives the maximum and minimum reading for the given ROI and the mean of the three minimum values across the three slices was used.

• **ADCmean**: Three to five circular ROIs of 50 mm^2^ were placed within the borders of the tumor on each axial slice, avoiding areas of cyst, necrosis or haemorrhage. A number of metastases show haemorrhage and the T1-, T2- weighted and b0 sequence were all assessed for evidence of intratumoral bleeding. Cases were also excluded at this stage if >50% of the tumor was affected by haemorrhage.

•**Peritumoral region**: The “peritumoral region” was taken to be the extent of signal abnormality on the T2-weighted sequence and “near” as <1 cm from the tumor edge (as assessed by the co-registered post-contrast T1-weighted sequence) versus “far” as ≥1 cm from the edge. Readings of the near (**ADCnear**) and far (**ADCfar**) peritumoral region were taken using three circular 50 mm^2^ ROIs on up to three on contiguous slices.

• Up to four control readings were taken from the unaffected contralateral white matter and a mean obtained. The above readings were normalised to this in order to minimise differences in acquisition parameters used at different institutions.

•**ATC**: To assess the metastasis-brain interface, the gradient of change of the ADC which we have called the “ADC Transition Coefficient” or ATC was calculated. As previously described [21] this involves placing four adjacent ROI (area: 22 mm^2^) in a line with the first inside the tumor boundary and the rest in brain. This was done along orthogonal trajectories across the anterior, posterior and lateral tumor tumor borders (avoiding cysts, necrosis and anatomical boundaries: ventricle, skull vault, cortex, falx cerebri). The gradient of a linear regression line (which provided the best fit) through the four ADC values of the four ROI was derived and all the trajectories averaged to generate the ATC.

### Follow up & statistical methods

Patients were followed up in regional oncology clinics and repeat MRI brain taken only if symptoms were present. Local recurrence or progression was defined as demonstrable brain disease on a post-operative MRI with appropriate clinical correlation and management; patients dying before that point were censored at the last clear brain scan (CT or MRI) or last clinical appointment when neurologically well. Patients who could not be confirmed deceased were censored at the last clinic appointment that had been recorded.

Data were analysed and graphs generated using Statistica version 6 (StatSoft, Inc. 2003) and SPSS version 20.0 (IBM co., 2011). For analysis of survival, the Kaplan-Meier product limit method was used and life tables with survival curves plotted. Log rank tests were applied to detect differences between paired groups and Wilcoxon modification (the Breslow chi square test) used in cases where hazard did not appear to be proportional. For multivariate analysis Cox regression was used. Standard tests were used for comparisons of means and analysis of variance and descriptive statistics, distributions were plotted for all variables to check assumptions. The level of significance was set at 95% to reject the null hypothesis and all p-values and confidence intervals are stated in the Results section.

## Results

### Clinical outcomes

The demographic details of the cohort of 76 patients are summarised in Table 1. The median survival from first diagnosis of cerebral metastasis to death was 9.3 months (range 0.9 – 34.3 months). 11 cases were censored, mainly due to death by other causes or loss of follow up suggesting the length of the follow up period was adequate. It was not appropriate to analyse local recurrence where the tumor was not fully resected; in the 66 cases where gross total resection was performed, local recurrence occurred in 16 patients (24%) at a median of 17.7 months from presentation (95% CI 10.9 – 24.6). The factors associated with overall and progression free survival, including standard MRI measures such as tumor size, volume and number, are summarised in Table 2.

**Table 1:** Demographics and treatment summary for the series (76 patients)

**Age at diagnosis/years (mean & range)**	**60.6 (35.9 – 81.4)**	
**Gender**	**36 male : 40 female**	
Size of operated lesion	27 (9 – 70)	Diameter/mm
(Median & range)	8.9 (0.3 – 130)	Volume/cc
***Disease information***	Number	%
Primary cancer type		
Non-small cell lung	36	47.4
Small cell lung	6	7.9
Breast	10	13.7
Melanoma	5	6.6
Gastrointestinal	5	6.6
Urogenital	4	5.3
Ovarian	4	5.3
Squamous cell tonsil	1	1.3
Unknown primary	5	6.6
Location of operated lesion		
Supratentorial	64	84.2
Infratentorial	12	15.8
Number of brain mets		
Solitary	56	73.7
2 lesions	11	14.5
>2 lesions	9	11.8
RPA class		
I	19	25
II	51	67.1
III	6	7.9
GPA score		
<1.0	6	7.9
1.5-2	20	26.3
2.5-3.0	39	51.3
>3.0	11	14.5
Neurosurgery		
Gross total resection	67	88.2
Partial resection/Biopsy	9	11.8
Adjuvant chemotherapy for systemic cancer	45	59.2
Whole brain radiotherapy after neurosurgery (30 Gy/5# as standard)	56	73.7

**Table 2:** Clinical and radiological factors associated with survival and progression

**Factor**	**Overall survival median/months**	**95% CI**	**Significance on univariate analysis**	**Progression free survival median/months**	**95% CI**	**Significance on univariate analysis**	
GPA score							
< 1.0	5.3	0.4 – 10.2	0.306	Not reached		0.936	
1.5-2	6.8	0 – 14.0			18.4		-
2.5-3.0	9.3	8.0 – 10.6			17.8		6.1 – 29.4
> 3.0	9.8	7.8 – 11.7			13.1		9.0 – 17.2
RPA class							
I	14.0	6.1 – 22.0		0.030*	17.7	14.6 – 20.9	0.636
II	8.4	5.1 – 11.8			13.1	9.7 – 16.6	
III	2.0	0 – 6.2			Not reached		
Primary cancer						(too many censored cases in each category for analysis)	
Lung non-small cell	9.2	2.0 – 19.8	0.061	Not reached			
Lung small cell	4.5	1.0 – 28.9			10.5		-
Breast	10.4	0.9 – 21.2			9.2		5.6 – 12.9
Colorectal	6.8	5.3 – 12.3			Not reached		
Melanoma	11.5	2.4 – 14.7			7.3		4.9 – 9.7
Urological	4.0	1.1 – 9.6			Not reached		
Ovarian	11.2	4.7 – 30			17.8	-	
Unknown	5.6	1.5 – 22.8			18.4	-	
Location							
Supratentorial	9.6	8.2 – 11.1	0.070	17.7	10.8 – 24.6	0.872	
Infratentorial	4.4	0 – 10.1			Not reached		
Surgery							
Biopsy	5.9	0 – 16.4	0.091				
Resection	9.6	8.1 – 11.0					
Adjuvant whole brain radiotherapy							
Yes	10.5	8.9 - 12	0.000*	17.7	10.9 – 24.6	0.000*	
No	2.4	1.5 – 3.3			Not reached		
Adjuvant chemotherapy							
Yes	10.5	8.2 – 12.7	0.001*	15.7	10.7 – 20.6	0.240	
No	5.3	3.0 – 7.6			Not reached		
Diffusion MR metrics							
ADCmin > 919 × 10^-6^ mm^2^/s	9.7	8.5 – 11.0	0.049*	18.4	13.2 – 23.6	0.087	
ADCmin < 919 × 10^-6 ^mm^2^/s	6.2	3.7 – 8.8			11.3	8.4 – 14.3	
ADCmean > 1148 × 10^-6^ mm^2^/s	9.7	7.9 – 11.5	0.093	18.4	11.6 – 25.1	0.039*	
ADCmean < 1148 × 10^-6 ^mm^2^/s	6.7	4.9 – 8.5			11.3	5.8 – 16.8	
ATC > 0.279	6.8	5.3 – 8.4	0.041*	11.3	8.2 – 14.4	0.072	
ATC < 0.279	11.2	8.3 – 14.0			Not reached		
Standard MRI measures							
Diameter > 30 mm	6.7	2.4 – 11.0	0.246	13.1	9.7 – 16.6	0.551	
Diameter < 30 mm	9.6	8.8 – 10.4			17.8	14.7 – 20.1	
Volume > 8.9 cc	6.7	1.9 – 11.5	0.077	13.1	9.2 – 17.1	0.158	
Volume < 8.9 cc	9.6	8.8 – 10.3			17.8	13.7 – 21.8	
Solitary	8.42	5.3 – 11.5	0.101	15.7	10.3 – 21.0	0.673	
2 lesions	14.1	10.7- 17.6			11.3	0.85 – 21.8	
> 2 lesions	6.1	5.1 – 12.2			Not reached		

Log rank tests were applied to detect differences between categories with Wilcoxon modification (the Breslow chi square test) in cases where hazard did not appear to be proportional. In all comparisons *indicates p < 0.05.

### ADC readings from the metastases & tumor cellularity

**Figure 2:**
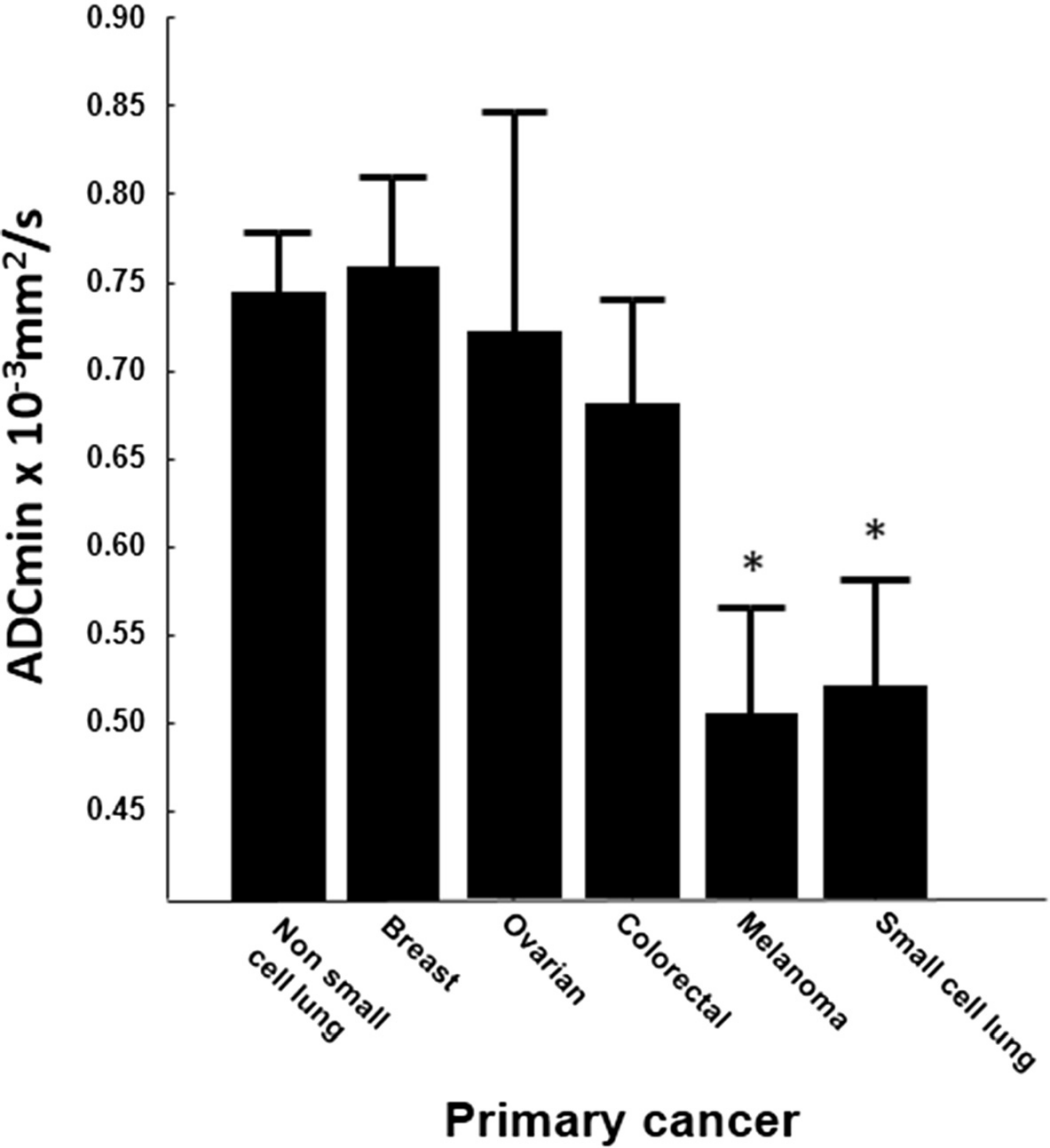
**ADC values of 76 brain metastases by primary cancer of origin.** The mean ADCmin value for each primary cancer type is shown +/- standard error and *indicates significant difference from the group at p < 0.05 by independent student t-test. All histological diagnoses were confirmed by neuropathology assessment after biopsy or resection and correlation with clinical data.

**Figure 3:**
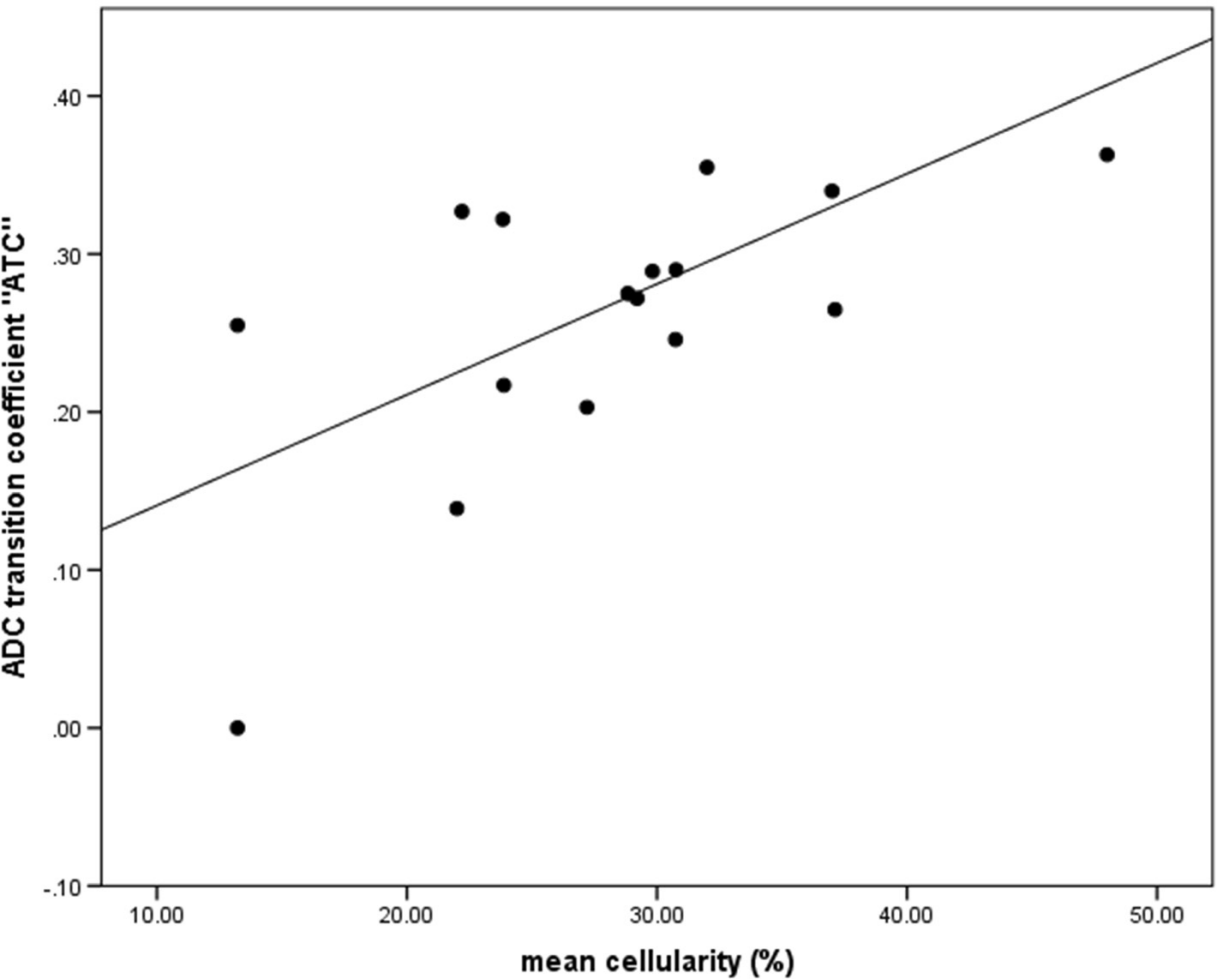
**The gradient in change of ADC across the tumor border on preoperative DWI for brain metastases, called the ATC (seeMethods) was strongly predictive of the cellularity, as shown in the scatterplot above.** Cellularity was assessed in a semi-automated fashion using ImageJ software from five different high power fields and ATC measured as shown in Figure 1. The line shows a simple linear regression model which was highly significant (ATC = 0.007* mean cellularity + 0.07, ANOVA F = 9.84, p < 0.001).

The median ADCmean for cerebral metastases in this series was 1148.1 × 10^-6^ mm^2^/s and the median ADCmin was 919.4 × 10^-6^ mm^2^/s. There were significant differences in ADC between cerebral metastases from different primary cancers by one-way ANOVA (F = 2.797, p = 0.025). On post hoc comparison, this was seen to be because metastases from the so-called “poorly differentiated” cancers such as melanoma (n = 5) and small cell lung carcinoma (n = 6) had a lower ADC compared to the metastases from carcinomas such as breast (n = 10), ovarian (n = 4) and colorectal (n = 4), as shown in Figure 2.Tissue was only available for 16 of 76 cases. Cellularity assessment by ImageJ software as compared to pathologist assessment appeared to be valid with consistency between the two observers (intraclass correlation coefficient = 0.61, p < 0.05). The mean cellularity was negatively correlated with both the ADCmin and the ADCmean and could be fitted to either of these with a simple linear regression model which was highly significant (for ADCmin, F = 7.99, p = 0.013 and for ADCmean F = 5.56, p = 0.033). ATC was strongly correlated with cellularity as shown in Figure 3 and was predicted by it using a simple linear regression model (F = 9.84, p = 0.007).

### Relation of tumor ADC to patient outcomes

**Figure 4:**
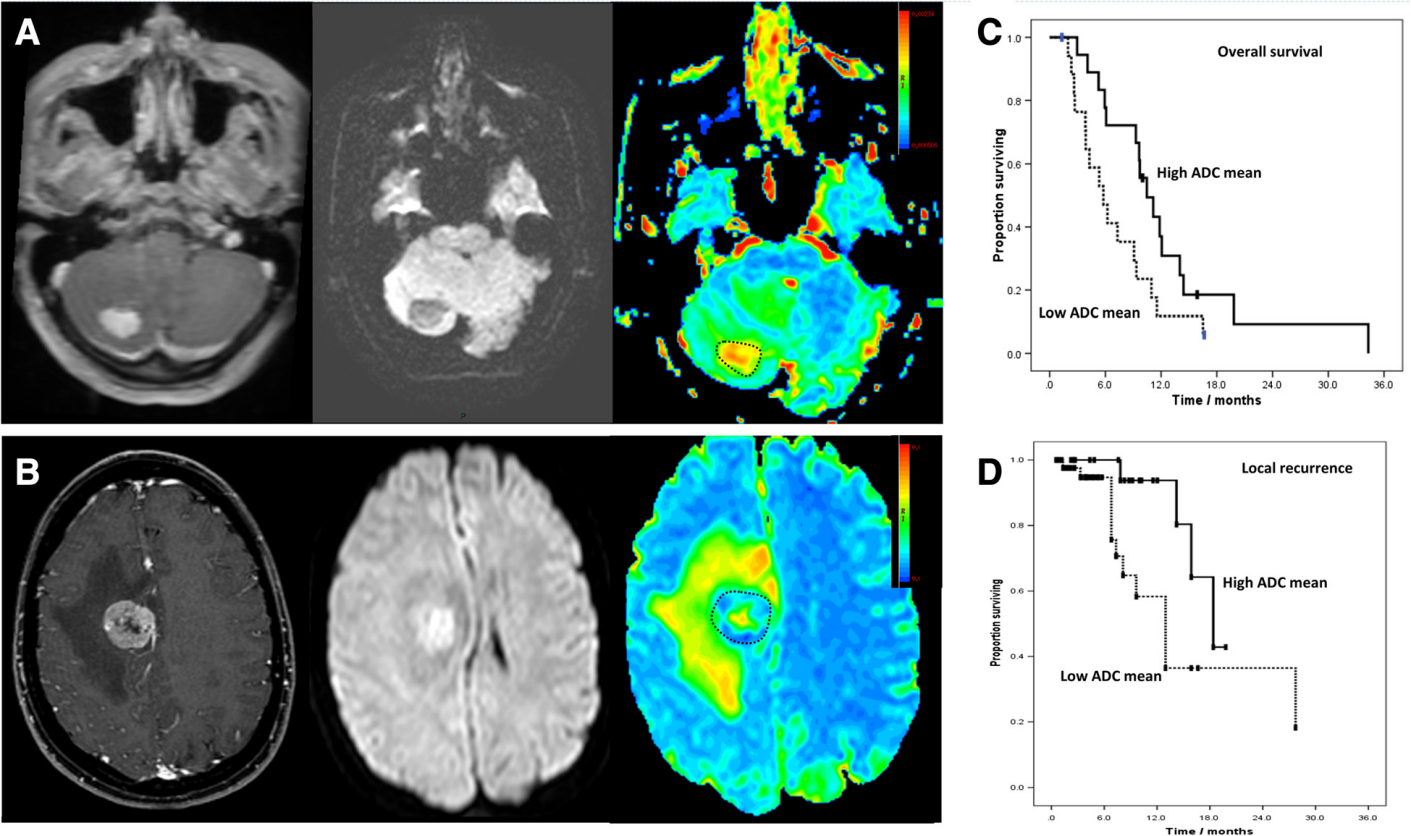
**ADC of the metastasis predicts subsequent patient outcomes.** Cases such as that in **A**. an enhancing lesion with hypo-intense DWI signal and higher than median ADC were compared to those such as **B**. an enhancing lesion but with hyper-intense DWI signal and corresponding low readings on ADC map. **C**. The largest group of metastases (n = 36) from a single primary - lung non-small cell carcinoma – were stratified into two groups by these readings of tumor ADC. Overall survival was significantly longer for cases with a high tumor ADCmean (10.5 months, CI 7.7 – 13.2) versus a low tumor ADCmean (5.8 months, CI 3.2 – 8.4, Log rank = 4.135, p = 0.042). **D**. For the 66 cases where gross total resection was performed, local recurrence in the brain occurred in 16. Metastases with a higher ADC showed significantly longer progression free survival, 18.4 months (95% CI 11.6 – 25.1) versus 11.3 months (95% 5.8 – 16.8) in those with a low ADCmean (Log rank test, Chi Square 4.263, p = 0.039).

For survival analysis, the patients were grouped based on each of the DWI characteristics of their cerebral metastases and the two groups compared. The cases having a higher than median ADCmin (>919.4 × 10^-6^ mm^2^/s) showed a longer overall survival, median 9.7 months (95% CI: 8.5 – 11.0) versus those with a lower ADCmin (median survival 6.2 months, 95% CI: 3.7 – 8.8, Breslow Chi square 3.87, p = 0.049). There was no difference in the proportion of patients receiving WBRT in the high versus the low ADCmin groups that would confound the effect on survival (Chi square = 3.49, p =0.062). The effect was also seen if the median ADCmean was used as the cutoff to define the two groups but was not statistically significant: OS 9.7 months, 95% CI 7.9 – 11.5 for patients with metastases having a higher ADCmean (>1148.1 × 10^-6^ mm^2^/s) versus 6.7 months for lower ADCmean, 95% CI 4.9 – 8.5, Breslow Chi square = 2.83, p = 0.093.Regarding local control, the median progression free survival was 11.3 months (95% 5.8 – 16.8) in those cases with a low ADCmean reading versus 18.4 months (95% CI 11.6 – 25.1) in those with a high ADCmean (Log rank test, Chi Square 4.263, p = 0.039). This is illustrated in Figure 4. This effect was also seen when the median ADCmin was used as the cutoff to define the two groups but did not reach statistical significance (high ADCmin, progression free survival 18.4 months, 95% CI 13.2 – 23.6 versus low ADCmin, progression free survival 11.3 months, 95% CI 8.4 – 14.3, Log rank = 2.93, p = 0.087).

Non-small cell lung cancer metastases represented the largest group of cases from a single primary cancer (although further detail was not known, even within this group there would be expected to be subdivisions such as adenocarcinoma and squamous cell carcinoma; it is at least more uniform than any previous analysis which includes multiple different primary sites) and this subgroup of 36 cases was therefore analysed separately in order to confirm that the ADC has value in and of itself as a prognostic marker not simply as a surrogate of the primary tumor type. Overall survival was significantly longer for non-small cell lung cancer cases with a high tumor ADCmin at 9.7 months (CI 6.7 – 12.6) versus 5.3 months (CI 2.2 – 8.5) for those with low ADCmin, Log rank Chi square = 4.008, p = 0.045. The same effect was observed and was significant using median ADCmean as the cutoff because higher ADC group had median OS 10.5months (CI 7.7 – 13.2) versus 5.8 months, (CI 3.2 – 8.4) for low ADC group, Log rank = 4.135, p = 0.042.

### ADC changes at the brain-metastasis interface

The change in diffusion across the brain-metastasis interface might be expected to relate to changes at the cellular level and hence even reflect tumor-stroma interactions. We have previously devised and applied a measure of the changing ADC at the metastasis-brain boundary, called the “ADC transition coefficient” or ATC. This could be calculated for 70 cases (as described in the Methods there needs to be a boundary to surrounding tissue to measure and calculate a reading) and found the ATC to have a median value of 0.279 (range 0.063 - 0.453). The ATC showed no relationship to the primary cancer type on one-way ANOVA (df 11, F = 0.348, p = 0.9) and weak negative correlation with ADCmean and ADCmin (Pearson correlation coefficient -0.39 and -0.33 respectively, p > 0.05) suggesting it was measuring a different feature rather than just acting as a surrogate of the other ADC characteristics or the primary cancer.

**Figure 5:**
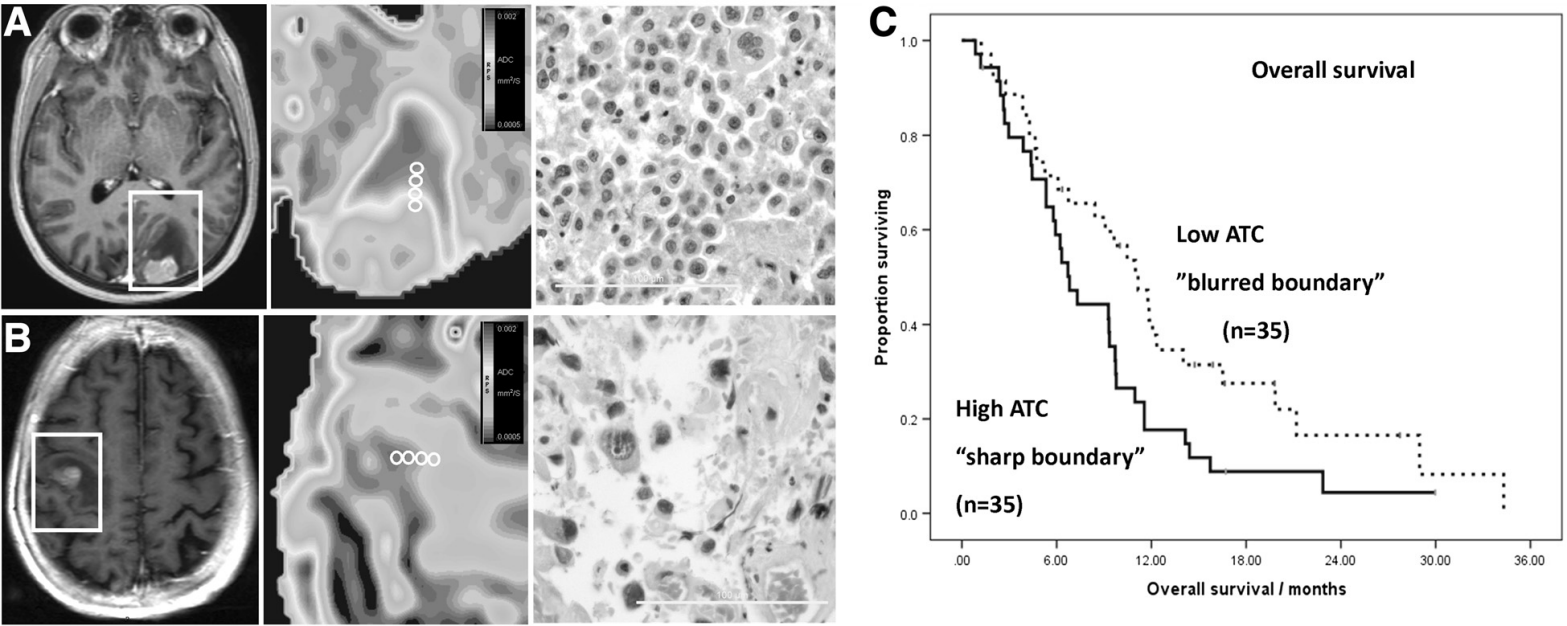
**Analysing the brain-metastasis interface.** Metastases could be stratified by the sharpness of the tumor border on ADC maps. Panel **A**. demonstrates a lesion with a high ATC or “ADC transition coefficient” implying a sharp border. This type of metastasis tended to have a high cellularity (H & E section x200). Panel **B**. demonstrates a case which looks superficially similar on postcontrast T1-weighted sequence but actually has a much more diffuse border on the ADC map and hence a low ATC, with lower cellularity. **C**. These metastases differ in their outcomes and the cases with a high ATC (> median) and therefore a sharp boundary had a shorter overall survival 6.8 months (95% CI 5.3 – 8.4) as compared to those with a low ATC and hence diffuse boundary (11.2 months, 95% CI 8.3 – 14.0, Log rank Chi-Square = 4.19, p = 0.041). This effect was significant even in multivariate analysis incorporating traditional clinical predictors.

The group was divided into two based on the ATC, and those patients with a metastasis showing high ATC (> median) and therefore a *sharp* boundary on the ADC map had a shorter overall survival 6.8 months (95% CI 5.3 – 8.4) as compared to those with a low ATC and hence diffuse boundary on ADC map (11.2 months, 95% CI 8.3 – 14.0, Log rank Chi-Square = 4.19, p = 0.041). This is illustrated more clearly in the conventional MRI scans and accompanying ADC maps in Figure 5. There was also a tendency to earlier local recurrence in high ATC cases versus low ATC but this was not statistically significant (shown in Table 2).

### ADC readings in the peritumoral region

Initially we did not find a large variability in the ADC around a given metastasis and for the 76 cases together, the mean “near” peritumoral ADC was 2028.4 × 10^-6^ mm^2^/s which did not differ significantly from the mean “far” peritumoral ADC of 1994.9 × 10^-6^ mm^2^/s. There was no significant variation of either variable by primary cancer type on one-way ANOVA. There was also no significant differences in patient outcomes (overall survival, local recurrence) when the cohort was divided into two “high” and “low” groups by either near peritumoral ADC, far peritumoral ADC or near:far ADC ratio and actuarial survival analysis performed.

**Figure 6:**
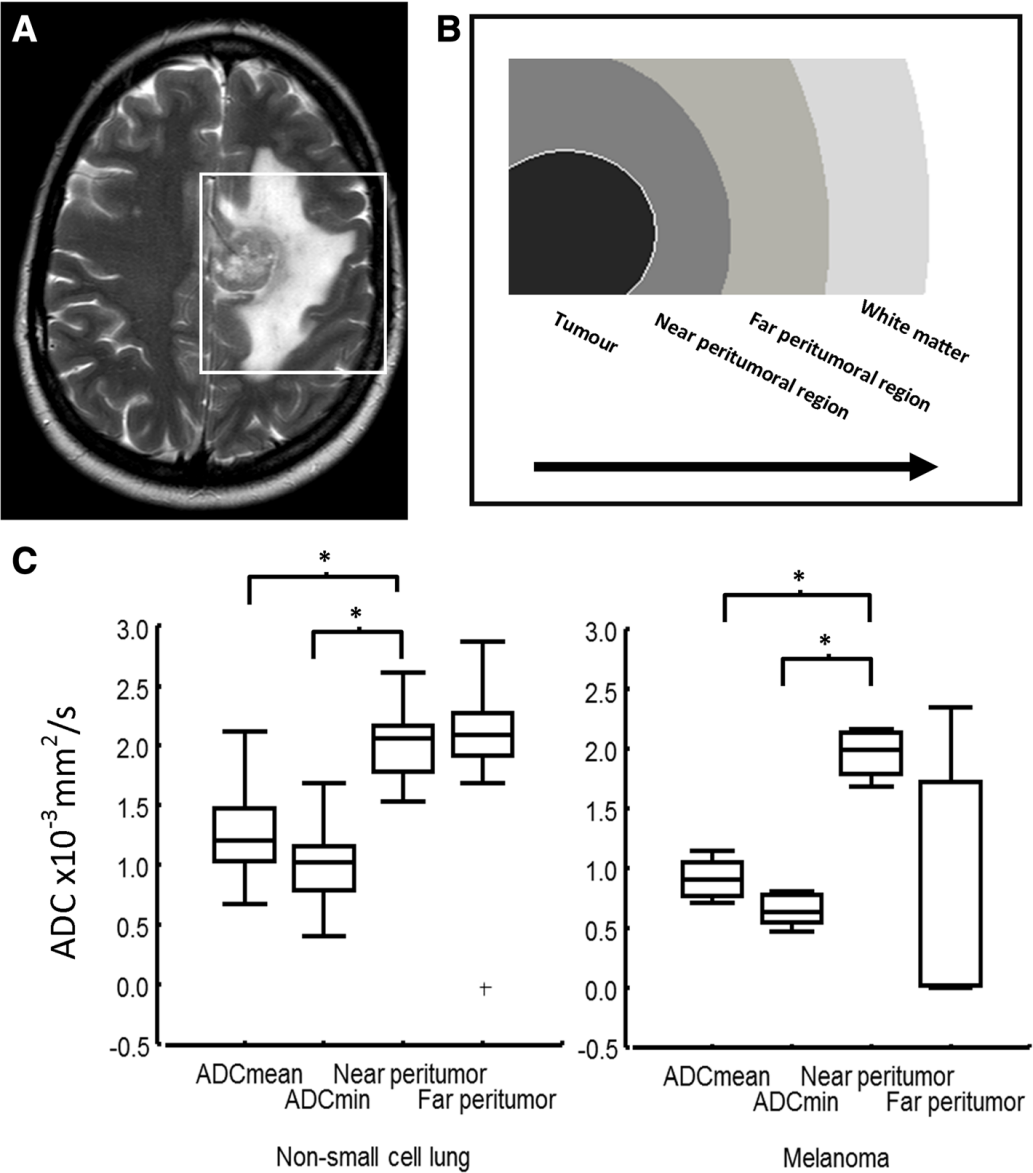
**Changes in diffusion around a brain metastasis.** A solitary metastasis in a patient with known renal cell carcinoma is shown in **(A)** with a clear boundary, necrotic centre and surrounding oedema, visible on the T2-weighted sequence. What is happening in the region around the metasasis? There is substantial evidence that this region consists of vasogenic oedema, higher diffusion and lower perfusion when compared to high grade glioma for example, but what about comparing *within* a group of metastases. **(B)**. One can place serial regions of interest and measure and plot the ADC changes from the necrotic centre of the tumor through the leading edge and into the peritumoral regions. **(C)** Box and whisker plots showing the median, interquartile range and outliers for ADC readings in and around melanoma (n = 5) and non-small cell lung cancer (n = 36) metastases demonstrates differences in this ADC signature between the two groups and within them but the biological correlate of these differences needs to be investigated.

When visually inspecting the ADC maps for the 76 cases, however, it appeared that for a minority of metastases, in particular those from melanoma primaries, diffusion *did* vary greatly between the near and far peritumoral regions. The values of ADC at each point going out from the tumor towards normal white matter were plotted for illustrative purposes to show the “ADC signature” for that type of metastasis and an example of this is given in Figure 6. It was relevant to directly compare such patterns of ADC change in two types of cancer known to show differing mechanisms of brain invasion, as outlined in Discussion. The ratio of the near and far ADC values were therefore calculated for metastases of melanoma and non-small cell lung cancer and it was seen that for melanoma metastases this ratio was significantly higher (independent 2-tailed t test = 2.259, df 36, p = 0.03).

### Multivariate analysis

A Cox regression model was generated using the radiological and non-radiological factors found to be significant discriminators of overall survival in univariate analysis. Only post-operative WBRT (hazard ratio 3.1, 95% CI 1.5 – 6.8, p =0.004) and low ATC (hazard ratio 0.54, 95% CI 0.3 – 0.97, p = 0.04) were found to be significantly associated with prolonged overall survival in this series. Multivariate analysis was attempted for progression free survival however modelling was inaccurate with large errors due to the high proportion of censored data (only 24% of the group or 16 cases developed local recurrence, as stated).

## Discussion

### The value of tumor ADC in cerebral metastases

This is the largest examination of diffusion-weighted MRI and cerebral metastases to date. The numerical values for tumor ADC are in line with previous investigations of cerebral metastases which would be expected given the standard methods and protocols used for imaging and analysis [6,7,10,13,14,22,23]. We used a variety of methods of taking measurements from the ADC maps so as not to miss an effect due to our measurement technique. We have confirmed that ADC varies between cerebral metastases from different primary cancers but like others not sufficiently to distinguish the individual primary cancer type [24,25]. This may be because the ADC reflects more general characteristics like cellularity. In support of this, ADC values were lower for the group of poorly differentiated metastases of melanoma and small cell lung cancer compared to the group of metastases from carcinomas (including breast and colorectal). We also corroborate the suggestion that tumor cellularity is inversely related to tumor ADC for a sample of our cases where tissue was available, whether measured by the minimum or the mean ADC. This is logical as more densely packed cells would broadly be expected to restrict diffusion, with the caveat that this coarse method of calculating ADC maps does not reflect the more subtle distinctions between intra and extracellular diffusion that others have derived using more advanced models in animals [26,27]. However it has also been shown in cerebral metastases *post mortem* that a dense extracellular matrix correlates with low ADC, similarly suggesting a role for the tumor-stroma interaction we discuss later in human cases [6].

Our survival analyses generally support the finding of this smaller study [6] that the pre-operative mean ADC of a cerebral metastasis is correlated with subsequent overall patient survival and we have now demonstrated this for a single tissue type; non-small cell lung carcinoma. We did not find that this effect persists in multivariate analysis. This may be due to including oligometastatic disease in this series but it seemed reasonable to include such cases as they all had a single larger, dominant lesion and we included the number of lesions as a confounding factor as it forms part of the GPA score. These findings that ADC predicts prognosis are in keeping with a number of reports from other brain tumors where DWI has been applied to predict stage and prognosis [28-30] as well as other organ systems such as renal, rectal, head and neck or cervical tumors [31-35]. In our series, in addition to whole brain radiotherapy - which is a treatment – it was only the *change* in diffusion across the tumor boundary which we call the ADC transition coefficient (ATC) that independently predicted the overall survival in multivariate analysis despite all other clinical factors. Why should this be?

### The brain-metastasis interface

We have shown that changes in ADC across the brain-metastasis interface can be used to define the “sharpness” of this boundary, yielding additional information compared to assessing the boundary on postcontrast T1-weighted sequences alone. Rather than a low ATC and diffuse boundary on ADC map being associated with poor survival - as one might expect if this simply represented a more infiltrative pattern of growth - the reverse was true. A high ATC may in fact reflect high cellularity at the leading edge of the metastasis and hence greater tendency to recur locally. In support of this, the ATC was strongly linearly related to tumor cellularity in a sample of cases where tissue was available. One might predict that these cases may benefit from more aggressive local adjuvant treatments, such as wider surgical margins or radiosurgery “boost” to the cavity. Previous work on the interface between cerebral metastases and the surrounding brain has been conducted using histological methods on surgically resected or *post mortem* specimens [36-38] and this has certainly suggested that there are different patterns and depths of invasion, even if this has not been related to clinical outcomes. Furthermore, it has been shown that wider resection margins may improve clinical survival [39] and that ADC changes across the border of another brain tumor - oligodendroglioma - may predict growth pattern and aggressiveness [21]. Novel therapies acting at the brain-metastasis interface are being developed [40], therefore more accurate non-invasive, radiological methods may be needed to assess the degree of invasion of cerebral metastases and characterise the tumor boundary changes seen at diffusion-weighted MRI.

### The peritumoral region

The MRI characteristics of the peritumoral region of cerebral metastases have previously been examined only in order to distinguish these tumors from glioma and abscess [15,41,42]. In this context the contrast enhancing margin has been taken as the reference and the near peritumoral region has been taken as being within 1 cm of this edge. We have shown that for some metastases the ADC varies considerably from the core of the tumor, toward the leading edge and out into both the near and far peritumoral regions. It is known that the extent of T2-weighted oedema (over 1 cm in width from the tumor edge +/- midline shift) may be a prognostic factor [43] and the different peritumoral ADC values in different metastases may be due to differences in the intensity of peritumoral oedema and/or underlying vascular permeability due to different patterns of infiltration. Melanoma metastases, for example, showed a significantly higher near:far peritumoral ADC ratio than non-small cell lung cancer metastases in this study. These tumors are known to show different patterns of growth [16]; melanoma metastases co-opt existing vessels whereas lung adenocarcinoma (the most common non-small cell lung cancer) stimulates neoangiogenesis and the pattern of peritumoral ADC changes might reflect this. In animal models peritumoral ADC changes are seen *before* postcontrast T1-weighted enhancement as micrometastasis formation and multiplication occurs [44]. Tissue samples with paired radiological data would be needed to confirm what the correlate of these ADC changes are in humans, however it is unlikely that one would be practically able to gather tissue from deep within the region of peritumoral oedema around a metastasis as has been done for glioma [45]. The brain-metastasis boundary is therefore a more fruitful area to investigate in the first instance. One could ask what the biological differences in pattern of infiltration, vascularity, protein expression and inflammatory response are around metastases with a sharp versus a diffuse ADC border and therefore could the ATC or similar measures of diffusion change be used as a surrogate of such biological activity.

### Limitations and future directions

A prospective study with image guided sampling in the course of routine resection would provide matched MRI and tissue data within and around cerebral metastases (as has been done with glioma and in animal models [44]) and should confirm the underlying biological changes which account for the ADC patterns seen. This was a retrospective study and therefore tissue was not universally available and was in paraffin embedded blocks from non-specific areas of the tumor, hence such an analysis was not possible. This study only examined ADC maps as more gradient directions were not available (again due to the MRI data having already been acquired and the analysis being retrospective) but ideally, more MRI sequences would be analysed pre-operatively including assessment of anisotropic diffusion, via fractional anisotropy. Disruption of white matter tracts is traditionally only seen in infiltrating tumors such as glioblastoma [46] and it would be important to observe if more infiltrative brain metastases as described pathologically [47] show different fractional anisotropy values in the peritumoral region. These findings are also cautioned by the need for validated, reproducible methods when measuring DWI metrics. In particular, we found differences in “tumor ADC” between measuring whole tumor values, minimum values and taking the mean of multiple ROIs. We have previously described good inter and intra-observer agreement measuring ADC metrics with various ROI methods in brain metastases on different post processing platforms [20]. A logical step would be to validate these metrics – in particular the ATC - on a separate, larger dataset and perhaps integrate MRI measures with conventional predictive models such as RPA and GPA.

Different MRI protocols from different vendors have had to be combined in this series due to a need for sufficient power for survival analysis. Again, data gathered in a prospective fashion on a single scanner with uniform protocols would be preferable. However ADC maps are theoretically and practically relatively resistant to variations in MR parameters (for example diffusion is insensitive to B_1_ errors). More sophisticated DWI parameters, including assessment of anisotropic diffusion (fractional anisotropy) which we would intend to study next, present more variation between centres. The use of phantoms and comparison of normal white matter readings may be needed to reduce and quantify between centre variation respectively [48]. The precise dosing and timing of corticosteroid therapy could also be established in a prospective series and effects on DWI parameters assessed.

The prevalence of intratumoral haemorrhage in many brain metastasis types is a problem and reduces the number of cases which are suitable for analysis (cases with overwhelming haemorrhage were excluded as stated in the Methods) as well as potentially making these findings less relevant to cases which tend to bleed such as melanoma and renal. Nonetheless a reasonable spread of common histological types was used here. Newer systemic therapies for treating cerebral metastases may lead to significant differences in survival between different tumor types and comprehensive clinical data with large numbers would be needed to stratify cases in future, for example by HER2 responsiveness in breast carcinoma metastases. This makes large scale biomarker studies practically difficult and the need for collaboration between centres paramount.

## Conclusions

Diffusion-weighted MRI demonstrates changes in the tumor, across the tumor edge and in the peritumoral region which may not be visible on standard MRI and this may be useful in predicting patient outcomes for cerebral metastases.

## Abbreviations

ADC: Apparent diffusion coefficient; ATC: Apparent diffusion coefficient transition coefficient; DWI: Diffusion-weighted imaging; H & E: Haematoxylin and eosin; GPA: Graded prognostic assessment; ROI: Region of interest; RPA: Recursive partitioning analysis; WBRT: Whole brain radiotherapy.

## Competing interests

The authors declare that they have no competing interests.

## Authors’ contributions

RZ, MR, MB and PR gathered data. KD, MR, MB, VS assisted and advised on image analysis. PR, VS and MDJ were involved in devising and supervising the work. RZ analysed data and wrote the final manuscript and all authors made substantial changes. All authors read and approved the final manuscript.

## Pre-publication history

The pre-publication history for this paper can be accessed here:

http://www.biomedcentral.com/1471-2342/14/26/prepub
